# Cardiopulmonary Fitness Correlates with Regional Cerebral Grey Matter Perfusion and Density in Men with Coronary Artery Disease

**DOI:** 10.1371/journal.pone.0091251

**Published:** 2014-03-12

**Authors:** Bradley J. MacIntosh, Walter Swardfager, David E. Crane, Nipuni Ranepura, Mahwesh Saleem, Paul I. Oh, Bojana Stefanovic, Nathan Herrmann, Krista L. Lanctôt

**Affiliations:** 1 Heart and Stroke Foundation Canadian Partnership for Stroke Recovery, Sunnybrook Research Institute, Toronto, Ontario, Canada; 2 Physical Sciences, Sunnybrook Research Institute, Toronto, Ontario, Canada; 3 Neuropsychopharmacology Research Group, Sunnybrook Research Institute, Toronto, Ontario, Canada; 4 Department of Medical Biophysics, University of Toronto, Toronto, Ontario, Canada; 5 Department of Clinical Pharmacology, University of Toronto, Toronto, Ontario, Canada; 6 Department of Psychiatry, University of Toronto, Toronto, Ontario, Canada; 7 Toronto Rehabilitation Institute, Toronto, Ontario, Canada; University of Jaén, Spain

## Abstract

**Purpose:**

Physical activity is associated with positive effects on the brain but there is a paucity of clinical neuroimaging data in patients with coronary artery disease (CAD), a cardiovascular condition associated with grey matter loss. The purpose of this study was to determine which brain regions are impacted by cardiopulmonary fitness and with the change in fitness after 6 months of exercise-based cardiac rehabilitation.

**Methods:**

CAD patients underwent magnetic resonance imaging at baseline, and peak volume of oxygen uptake during exercise testing (VO_2Peak_) was measured at baseline and after 6 months of training. T1-weighted structural images were used to perform grey matter (GM) voxel-based morphometry (VBM). Pseudo-continuous arterial spin labeling (pcASL) was used to produce cerebral blood flow (CBF) images. VBM and CBF data were tested voxel-wise using VO_2Peak_ and age as explanatory variables.

**Results:**

In 30 men with CAD (mean age 65±7 years), VBM and CBF identified 7 and 5 respective regions positively associated with baseline VO_2Peak_. These included the pre- and post-central, paracingulate, caudate, hippocampal regions and converging findings in the putamen. VO_2Peak_ increased by 20% at follow-up in 29 patients (t = 9.6, df = 28, p<0.0001). Baseline CBF in the left post-central gyrus and baseline GM density in the right putamen predicted greater change in VO_2Peak_.

**Conclusion:**

Perfusion and GM density were associated with fitness at baseline and with greater fitness gains with exercise. This study identifies new neurobiological correlates of fitness and demonstrates the utility of multi-modal MRI to evaluate the effects of exercise in CAD patients.

## Introduction

Aerobic exercise not only reduces cardiovascular risk but also affects the brain by increasing angiogenesis, neurogenesis and synaptogenesis [1]. Animal studies have identified brain regions that respond to exercise, including angiogenesis-related processes in motor circuits in rats [2], [3], mature aged monkeys [4], as well as the mouse hippocampus [5]. Replication of these findings in human studies has been limited to date, and performed primarily in healthy cohorts. For example, healthy older adults participated in a 12 month walking intervention and this contributed to increasing the volume of the hippocampus [6]. Cross-sectionally, highly active older adults have increased perfusion in the precuneus region compared to age-matched sedentary adults [7]. Peak volume of oxygen uptake (VO_2Peak_), a measure of the capacity to transport and use oxygen during exercise, is associated with increased grey matter volume in multiple brain regions among older healthy adults. The regions include the anterior cingulate, inferior frontal gyrus and superior temporal gyrus, as reported by others [8]. Aside from the hippocampus, subcortical grey matter regions are typically not reported in human exercise neuroimaging literature, despite evidence from the animal studies that exercise impacts the basal ganglia [9], [10]. These studies and compelling reports on Alzheimer's patients [11], [12] provide the impetus to further characterize exercise-related effects on the brain in older clinical populations at risk for cognitive decline [13]. Cardiovascular and/or cerebrovascular patients are likely to garner significant benefits [14] and they are therefore the focus of the current study.

Coronary artery disease (CAD) involves intraluminal narrowing of the arteries that supply blood to the heart and it is associated with a cluster of vascular risk factors such as hypertension, dyslipidemia, history of smoking, increased central adiposity and sedentary behaviour. These factors have been variably linked with grey matter loss [15]–[17] and posited to contribute to brain hypoperfusion [18]. Importantly, VO_2Peak_ is a strong predictor of cardiac and all-cause mortality in CAD patients [19]. Exercise-based cardiac rehabilitation is thus indicated for the secondary prevention of cardiovascular events. The cardiopulmonary exercise test (CPET) is used to quantify VO_2Peak_, a highly reproducible objective measure of cardiopulmonary fitness. Clinically, the VO_2Peak_ is used to assess the efficacy of exercise interventions. Although increasing VO_2Peak_ is linearly related to a decreased risk of cardiovascular mortality [20], individual responses to exercise interventions can vary considerably.

In the current study, VO_2Peak_ is used to explain within-cohort variance seen on two magnetic resonance imaging (MRI) techniques: 1) cortical and subcortical grey matter (GM) density using voxel based morphometry (VBM) and 2) cerebral blood flow (CBF) using whole brain pseudocontinuous arterial spin labeling (pcASL). VBM is an ideal structural analysis technique to study both cortical and subcortical grey matter. Previous neuroimaging studies in healthy adults have found GM density in both cortical [8] and subcortical [6] regions to be correlated with exercise. pcASL is a sensitive technique that can provide blood flow measures that complement structural imaging [21]. In the present study, it is hypothesized that VO_2Peak_ will be correlated with increased perfusion and grey matter density in distinct brain regions in patients with CAD. In addition, it is hypothesized that baseline grey matter perfusion and density in these regions will predict changes in VO_2Peak_ over the course of an exercise intervention.

## Methods

### Participants

This study was approved by Sunnybrook and University Health Network research ethics boards. Participants entering a cardiac rehabilitation program were approached to participate in this study. Seventy participants were screened, 58 showed evidence of CAD, 42 were willing to be contacted by study personnel of which 10 were excluded (see below) and 32 provided written informed consent. Two participants were excluded due to poor quality MRI resulting in 30 patients for analysis in study. Due to the 4.5 to 1 bias of men CAD patients entering cardiac rehabilitation compared to women CAD patients [22] and established sex differences in cerebral blood flow [23],[24], male sex was an inclusion criteria for this study. Other inclusion criteria included age 55–80 years, a documented history of CAD: myocardial infarction (MI), narrowing of at least one major coronary artery, percutaneous coronary intervention (PCI), or coronary artery bypass graft surgery (CABG). Patients were excluded if they had contraindications to an MRI or any neurodegenerative disorder. In addition to cardiac history, demographic information, concomitant medications, body mass index (BMI), and histories of hyperlipidemia, diabetes mellitus, hypertension and smoking were ascertained.

### Cardiopulmonary exercise test

Cardiopulmonary fitness was assessed using a cycle ergometer (Ergoselect 200P, Ergoline, Bitz, Germany) symptom-limited graded exercise test at baseline and after 6 months of exercise. Workload was increased by 16.7 W every minute. Breath-by-breath gas samples were collected and averaged over a 20-second period using a calibrated metabolic cart (Vmax Encore, SensorMedics, Yorba Linda, CA) [25]. The peak volume of oxygen uptake per minute (VO_2Peak_) was calculated after dividing by the patient's mass to obtain VO_2Peak_ in units of mL/kg/min. MRI was performed within 1 month of CPET and within 2 weeks of beginning exercise.

### Cardiac rehabilitation exercise program

Cardiac rehabilitation consisted of aerobic and resistance training in a group setting under the supervision of exercise and medical specialists. Patients attended supervised exercise visits that included an aerobic walk or walk/jog once per week for 24 weeks. The 6-month cardiac rehabilitation program was at no cost to the participants due to national healthcare coverage and they received no remuneration to participate in the study. Patients were also expected to exercise five out of seven days of the week at home and document the duration, intensity and frequency of the exercise in weekly exercise diaries, which were monitored every week for compliance by an assigned exercise supervisor. Previously, we have reported on the efficacy of this program [26], and established that compliance is high in this population [27]. Patients were provided with nutrition documentation at the start of the program during education classes, but no formal diet was recommended/undertaken. Initial exercise prescription was a walking distance of approximately 1.6 km at an intensity equivalent to 60% of VO_2Peak_. Prescriptions progressed every 2 weeks to a maximum of 6.4 km and then to a maximum intensity of 80% of VO_2Peak_ as estimated from maximum heart rate measurements. Prescriptions did not exceed a maximum daily duration of 60 minutes.

### Magnetic resonance imaging

Neuroimaging was performed on a 3 Tesla MRI system (Discovery MR750, General Electric Healthcare) and using a body radio frequency (RF) coil for transmission and an 8 channel phased array RF head coil for signal detection. Structural imaging included: 1) high resolution T1-weighted data using 3D spoiled gradient recalled echo (TR/TE/TI = 8.1/3.2/650 ms, flip angle = 8deg, acquisition matrix 256×192×186, nominal spatial resolution 0.9×0.9×1 mm), 2) fluid attenuated inversion recovery (FLAIR) sequence (TR/TE/TI = 9700/141/2200 ms, flip angle = 90deg, acquisition matrix 256×192×48, nominal spatial resolution 0.9×0.9×3 mm) and 3) dual proton density, T2-weighted images (TR/TE1/TE2 = 2500/11/90 ms, flip angle = 90deg, acquisition matrix 256×192×48, nominal spatial resolution 0.9×0.9×3 mm). FLAIR images were used to enable automatic identification and masking of white matter hyperintensity (WMH) voxels that would otherwise influence GM density estimates on the VBM analysis. Proton density images were used to extract the brain from head. These latter two considerations are part of Lesion Explorer software, described elsewhere [28].

Perfusion weighted images were acquired using a pseudo-continuous arterial spin labeling (pcASL) sequence that was developed in the laboratory [29], performed with a labeling duration of 1500 ms and a post label delay of 1700 ms [30]. The labeling plane was prescribed with the help of time-of-flight angiography images at the level of or just superior to the carotid bifurcation. Labeling was typically done at the level of the 2nd cervical vertebrae where internal carotids and vertebral arteries run parallel to one another. Twenty-five control and tag images were acquired sequentially in the axial plane using TR/TE/Flip angle  = 4000 ms/17 ms/90deg and single shot echo planar imaging (EPI) readout. Seventeen slices were collected with gap of 1.4 mm, slice thickness of 4.2 mm and nominal voxel dimensions of 3.4 by 3.4 by 5.6 mm^3^. The pcASL volume was planned based on maximum coverage of cerebrum. In practise this meant that the cerebellum and superior portion of the cerebrum were not covered consistently.

### Post processing

Voxel based morphometry (VBM) was performed in FMRIB Software Library (FSL), with additional customized steps to account for white matter changes: 1) non-brain regions on T1 images were identified using brain extraction tool (BET), 2) FLAIR images were co-registered to the T1, 3) WMH masks were warped to T1, 4) voxels within WMH regions were replaced with intensity values equivalent to mean healthy WM along with Gaussian noise, thereby creating a flat intensity profile over the WMH region to ensure proper GM segmentation, and 5) grey matter estimates were generated in standard space using the standard FSL-VBM processing pipeline with a 4.6 mm full-width half-max Gaussian smoothing kernel [31].

ASL images were processed using FMRIB Software Library (FSL) tools. Post processing of ASL data included: perfusion-weighted difference images, motion correction and spatial smoothing by a Gaussian kernel of 5 mm full width at half maximum using “asl_preproc” available in FSL. CBF images were intensity normalized to a global level of 40 ml/100 g/min [32] and co-registered to a standard space atlas using affine registration. The CBF intensity normalization was done to reduce the between subject variance and thereby increase the sensitivity to the VO_2Peak_ effect of interest. Others report an increase in sensitivity from this normalization in clinical pcASL cohort studies [33].

### Statistics

VO_2Peak_ data were tested for normality in R (www.R-project.org) using the Shapiro test. Voxel-wise group analyses were performed using a general linear model whereby VO_2Peak_ was the explanatory variable of interest and age was included as a covariate. Statistical maps were calculated to determine voxels with a positive association of VO_2Peak_ on the MRI data. Images were reformatted to 3 mm isotropic voxels in standard space to ensure consistency between the two modalities. Randomise in FSL was used with 5000 permutations to characterize the null distribution of the data empirically [34]. Correction for multiple comparisons was performed using a two step procedure: 1) an evaluation of the false discovery rate (FDR) using the FSL program called FDR with a one way q = 0.05 followed by 2) a cluster level threshold of contiguous voxels with a minimum volume of 8 voxels (.22 mL) using the program 3dclust in AFNI. A secondary group analysis was performed on the baseline ASL and VBM data using the change in VO_2Peak_ (i.e. VO_2Peak_ at follow-up minus VO_2Peak_ at baseline). The same multiple comparison corrections were used in this case for the CBF and GM data. Finally, linear regression analyses were performed in R using change in VO_2Peak_ and age as independent variables of the baseline MRI data. These analyses were restricted to the areas identified by the cross-sectional findings.

## Results

### Baseline and change in VO_2Peak_

**Figure 1:**
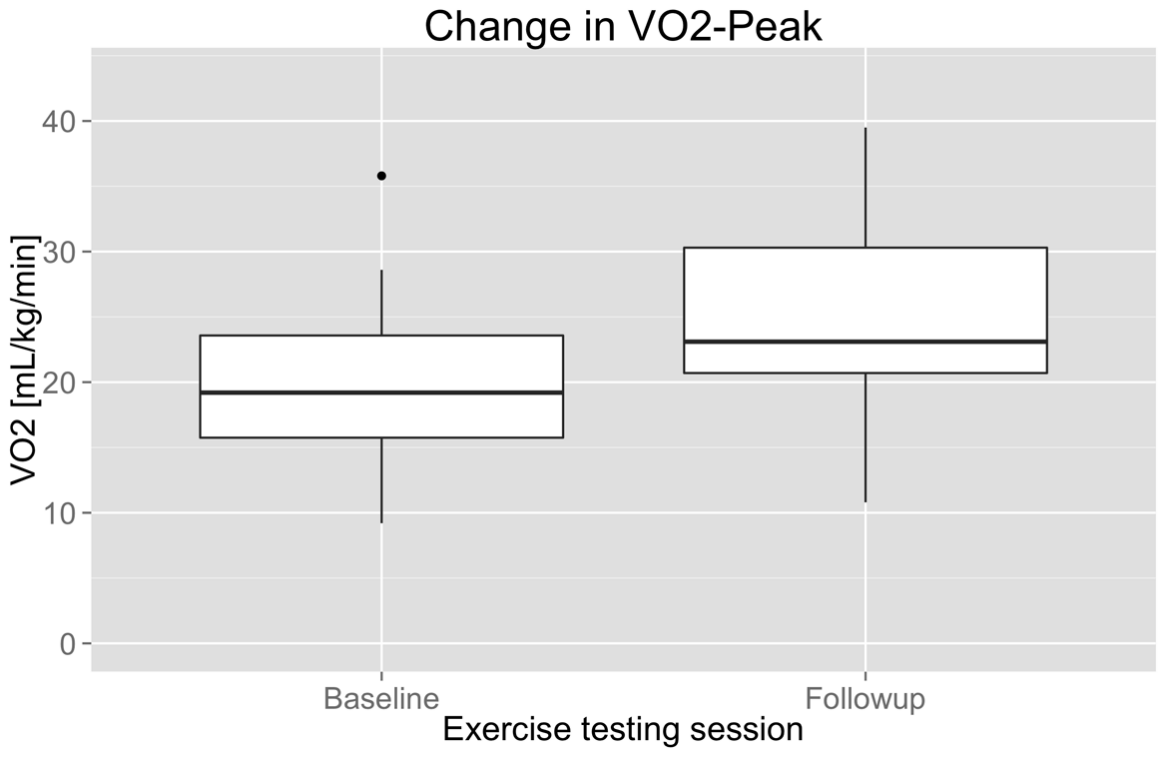
Mean cardiopulmonary fitness at baseline (N = 30) and after 6 months of exercise intervention (n = 29). The paired comparison shows a significant session effect (t = 9.6, df = 28, p<0.0001).

Table 1 shows baseline participant demographics. The time since most recent hospitalization for an acute coronary syndrome or intervention was 11.1 weeks (range 6.86–13.43). Twenty-nine out of 30 participants completed 6 months of cardiac rehabilitation and returned for follow-up CPET VO_2Peak_ testing. All 29 patients were compliant with cardiac rehabilitation protocols as assessed by their case manager based on exercise logs, attendance and fitness assessments. At baseline, the mean VO_2Peak_ was 20.5±5.9 mL/kg/min, which is 16% below the age-adjusted norm [35]. At follow-up, the mean VO_2Peak_ was 24.7±6.7 mL/kg/min among the 29 completers, 4% above the norm and significantly higher than baseline (t = 9.6, df = 28, p<0.0001; Figure 1).

**Table 1:** Participant Demographics (BMI  =  body mass index, DBP  =  diastolic blood pressure, SBP  =  systolic blood pressure, CABG  =  coronary artery bypass graft, MI  =  myocardial infarction, ASA  =  acetylsalicylic acid, ACE  =  angiotensin-converting-enzyme).

Demographics		Mean±SD or %
Age [years]		65.0±7.0
Education [years]		16.9±3.1
Marital status [partnered]		90%
Ethnicity [number, %]		
	Caucasian	27 (87%)
	South Asian	2 (6.5%)
	African American/Afro-Caribbean	2 (6.5%)
Employment status [number, %]		
	Employed	14 (45%)
	Not working/retired	17 (55%)
Vascular risk factors		
	BMI [kg/m^2^]	27.9±3.8
	Body Fat [%]	25.7±5.7
	DBP [mmHg]	73.4±7.9
	SBP [mmHg]	124.3±15.2
	Hypertension	40%
	History of smoking	53%
Cardiac history		
	Stent	47%
	CABG	47%
	MI	37%
Concomitant medications		
	ASA use	100%
	Statin	97%
	Beta-blocker	73%
	ACE inhibitor	57%

### Effect of VO_2Peak_ and baseline grey matter perfusion and density

**Figure 2:**
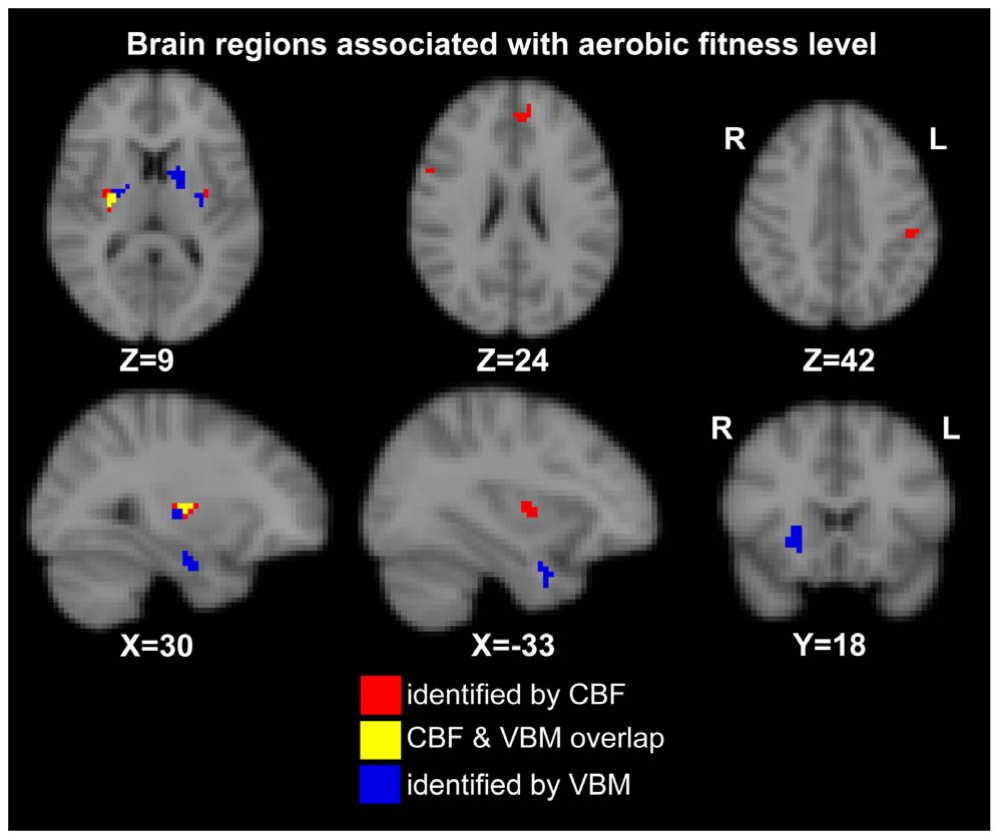
Voxel-wise analyses for CBF and GM density. Brain regions shown in color are significantly correlated with baseline VO_2Peak_ after controlling for age and correcting for multiple comparisons. CBF voxels are shown in red; VBM voxels are shown in blue; the region in yellow is the right putamen and found to be overlapping for CBF and GM data.

Figure 2 shows the brain regions that were identified voxel-wise as significantly positively correlated with VO_2Peak_ after accounting for age as a covariate. Significant voxels for the CBF data are shown in red and included bilateral putamen, left anterior cingulate, right premotor cortex and the left postcentral gyrus regions. Significant voxels for the VBM are shown in blue and included bilateral putamen, left caudate, right hippocampus, left temporal pole and right planum temporale. Voxels in the left and right bilateral putamen were detected by both VBM and CBF modalities (Figure 2; shown in yellow). Significant voxel volumes, i.e. cluster sizes, and Montreal Neurological Institute coordinates are listed in Table 2.

**Table 2:** Brain regions identified by the voxel-wise CBF and grey matter density analyses.

		MNI Coordinates				Baseline VO_2Peak_ model				Change in VO_2Peak_ model			
	Brain Region	# Voxels	X	Y	Z	adj R^2^	t-stat	p-value	sig	adj R^2^	t-stat	p-value	sig
**CBF**													
1	Putamen, left	23	−30	9	3	0.30	3.37	0.0023	**	−0.01	0.26	0.3999	
2	Putamen, right	16	30	−9	9	0.24	3.14	0.0041	**	−0.05	−0.26	0.4004	
3	Anterior cingulate, left	12	−3	42	24	0.34	3.65	0.0011	**	0.02	−0.35	0.3659	
4	Premotor cortex, right	9	51	9	27	0.24	2.78	0.0097	**	0.03	0.61	0.2751	
5	Postcentral gyrus, left	8	−48	−27	42	0.42	3.42	0.0020	**	0.30	1.78	0.0430	*
**VBM**													
1	Planum temporale, right	46	42	−15	−9	0.62	6.64	0.0000	**	0.00	0.00	0.5000	
2	Temporal pole, left	26	−33	3	−36	0.51	5.47	0.0000	**	0.00	0.66	0.2580	
3	Hippocampus, right	22	21	−21	−12	0.54	3.99	0.0005	**	0.26	−0.10	0.4599	
4	Caudate, left	20	−15	−3	6	0.64	4.31	0.0002	**	0.40	0.94	0.1778	
5	Putamen, right	18	21	18	−9	0.42	3.82	0.0007	**	0.26	2.24	0.0170	*
6	Hippocampus, right	12	33	−9	−24	0.60	4.35	0.0002	**	0.32	−0.63	0.2664	
7	Putamen, left	12	−30	−15	6	0.42	4.42	0.0001	**	0.00	−0.12	0.4541	

The number of voxels and MNI coordinates are listed. These regions were then used in a linear regression model to assess the effect of VO_2Peak_ and change in VO_2Peak_ with age as a covariate. * denotes significant at P = 0.05 on a 1-tailed test and ** denotes 2-tailed test.

**Figure 3:**
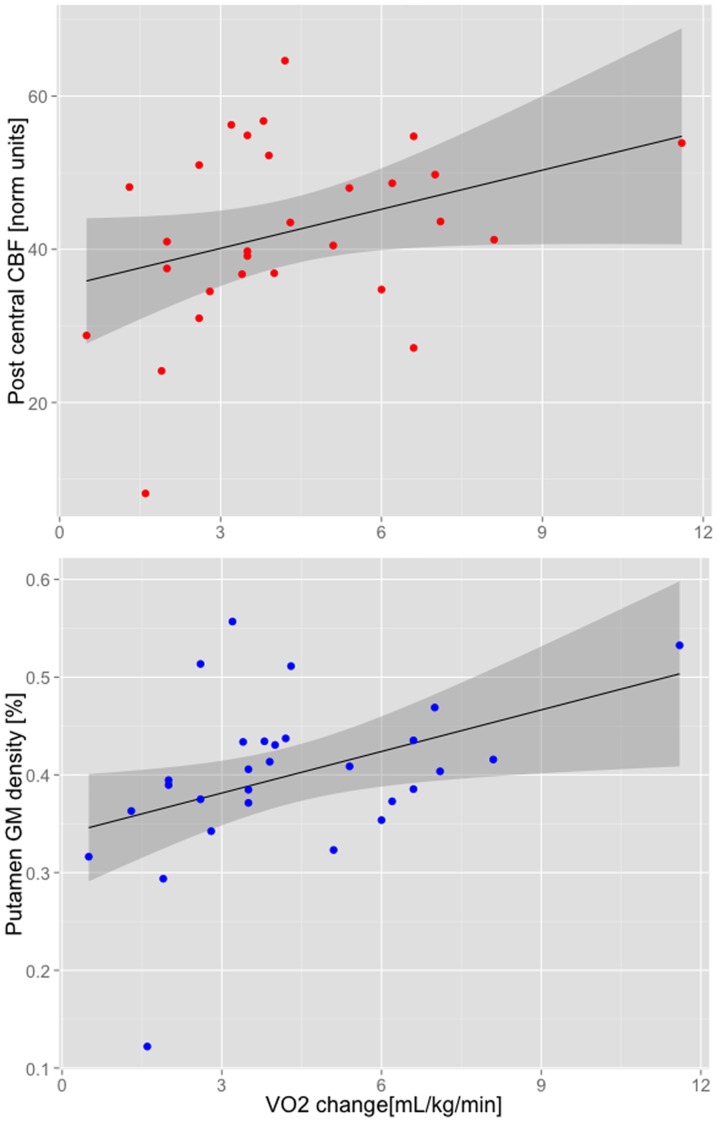
Scatter plots show MRI findings at baseline versus the change in VO_2Peak_. The linear regression analyses for these two regions / measures were significant (P<0.01). The line of best fit is shown with the grey shaded region showing the 95% confidence interval.

Voxel-wise analysis on the change in VO_2Peak_ data did not produce any significant voxels after multiple comparison correction. Linear regression analyses however found two significant brain regions from Figure 2 that were significantly related to the change in VO_2Peak_ (i.e. follow-up minus baseline) for a 1-tailed test at P = 0.05 (see Table 2). Scatter plots for CBF in the left post-central gyrus and the GM density in the right putamen versus change in VO_2Peak_ are shown in Figure 3.

## Discussion

This study demonstrates that cardiopulmonary fitness (VO_2Peak_) is positively associated with regional cerebral blood flow and grey matter hypertrophy in specific regions among adults with CAD. One striking finding was the localization of an exercise effect in the putamen, with converging evidence provided by both CBF and VBM analyses. The present multi-modal approach provides complementary structural and perfusion findings, illustrating that CBF was uniquely associated with cardiopulmonary fitness in cortical structures like the sensorimotor and premotor cortices, as well as the anterior cingulate. By contrast, cardiopulmonary fitness was associated with increased GM density in subcortical structures, the hippocampus, caudate and temporal regions, which are brain regions previously identified in studies of healthy adults[6]–[8].

Within the striatum, the results of the current study identified the putamen in both the ASL and VBM datasets, and the caudate in the VBM dataset as being positively associated with VO_2Peak_. These results are not altogether unexpected as they parallel the animal exercise literature For instance, McCloskey et al. used an optical method to show increased cytochrome oxidase metabolism in hindlimb and forelimb motor cortices and striatum due to chronic exercise [9]_ENREF_22. In another study, rats that underwent treadmill training showed a similar pattern of regional changes as seen by functional activation, namely basal ganglia, cerebellum, thalamus, and sensorimotor cortex [10].The putamen is known to receive motor pathway connections and is implicated in motor learning, while the caudate receives dorsolateral prefrontal pathway connections and is implicated in learning, feedback and reward [36].

The current study provides new evidence that brain measures prior to starting an exercise intervention can predict the change in fitness. As reflected in Table 2, it was putamen grey matter density and sensorimotor CBF at baseline predicted greater increases in VO_2Peak_ over the course of this exercise interventions. While much emphasis has been placed on understanding the effects of exercise on the brain, less has been done to establish neurobiological markers that predict who will benefit from an exercise program. These data add to limited and emerging literature suggesting that brain function can predict the effectiveness of exercise interventions [37], [38] and the current study suggests that these phenomena may have a quantifiable neurobiological basis. Further efforts will be needed to translate these preliminary findings into strategies to predict and improve outcomes.

This study has limitations, such as a relatively small sample size. In addition a non-CAD control group would have helped to establish whether the observed brain associations would generalize to a non-clinical cohort. Having said this the imaging methodologies were sensitive enough to detect associations with adequate power. Only men were considered for this study due to the preponderance of male participants in cardiac rehabilitation and the possibility that including a relatively small proportion of women might introduce heterogeneity in a small sample; therefore, the results cannot be generalized to women. Moreover, apart from gender, although the demographics of the included participants are characteristic of those who undertake cardiac rehabilitation, the study may have been subject to bias based on patterns of referral and intake into cardiac rehabilitation, further reducing generalizability. Although the study identifies a temporal relationship between VO_2Peak_ and grey matter measures, follow-up MRI was not performed, precluding our ability to establish a direct causation. Finally, the present study does not elucidate relationships between perfusion at rest and perfusion during exercise [39], which remains an important area for further exploration.

The association between putamen volume and VO_2Peak_ in this study of older men with CAD concurs with that reported recently in adolescents [40], identifying a consistent correlate of fitness throughout the human lifespan. Moreover, in the present study, larger right putamen volumes were associated with larger changes in VO_2Peak_ associated with the exercise intervention. The findings would be consistent with the involvement of the putamen in mediating changes to the dopaminergic reward system in response to exercise [41] and with a role of the putamen in initiating physical activity behaviours based on a history of reward [42]. These findings, taken together with the putamen's pivotal position in the striatal circuitry controlling motor function, implicate the putamen as a critical node in the relationship between brain and behaviour. Sensorimotor CBF was associated with baseline VO_2Peak_ and change in VO_2Peak_, which to our knowledge is a unique clinical finding to date and aligns with primate work showing exercise increases motor cortex vascular density [4].
